# Defective Differentiation of Adipose Precursor Cells from Lipodystrophic Mice Lacking Perilipin 1

**DOI:** 10.1371/journal.pone.0117536

**Published:** 2015-02-19

**Authors:** Ying Lyu, Xueying Su, Jingna Deng, Shangxin Liu, Liangqiang Zou, Xiaojing Zhao, Suning Wei, Bin Geng, Guoheng Xu

**Affiliations:** Department of Physiology and Pathophysiology, School of Basic Medical Sciences, Peking University, Beijing, 100191, China; The University of New South Wales, AUSTRALIA

## Abstract

Perilipin 1 (Plin1) localizes at the surface of lipid droplets to regulate triglyceride storage and hydrolysis in adipocytes. Plin1 defect leads to low adiposity in mice and partial lipodystrophy in human. This study investigated the roles of Plin1 in adipocyte differentiation. Plin1 null (-/-) mice showed plenty of multilocular adipocytes and small unilocular adipocytes in adipose tissue, along with lack of a subpopulation of adipose progenitor cells capable of in vivo adipogenesis and along with downregulation of adipogenic pathway. Before initiation of differentiation, adipose stromal-vascular cells (SVCs) from Plin1-/- mice already accumulated numerous tiny lipid droplets, which increased in number and size during the first 12-h induction but thereafter became disappeared at day 1 of differentiation. The adipogenic signaling was dysregulated despite protein level of PPARγ was near normal in Plin1-/- SVCs like in Plin1-/- adipose tissue. Heterozygous Plin1+/- SVCs were able to develop lipid droplets, with both the number and size more than in Plin1-/- SVCs but less than in Plin1+/+ SVCs, indicating that Plin1 haploinsufficiency accounts for attenuated adipogenesis. Aberrant lipid droplet growth and differentiation of Plin1-/- SVCs were rescued by adenoviral Plin1 expression and were ameliorated by enhanced or prolonged adipogenic stimulation. Our finding suggests that Plin1 plays an important role in adipocyte differentiation and provides an insight into the pathology of partial lipodystrophy in patients with Plin1 mutation.

## Introduction

Perilipin-1 (Plin1) is the first identified member in the perilipin family that are loosely grouped by sequence similarity of the first ~100 amino-acid terminal residues [1,2]. Plin2 to Plin5 that associate with lipid droplets but also exist in cytosolic compartments in various types of cells, and their functions are largely unknown. By contrast, Plin1 localizes only at the surface of lipid droplets exclusively in adipose and steroidogenic cells [2,3]. Plin1 constitute ~0.25% of total protein in adipocyte [3] and plays fundamental roles in stabilizing lipid droplets and controlling lipolysis [2]. Native Plin1 may act as a barrier to prevent triglyceride hydrolysis by lipase, thus enhancing lipid droplet formation [2,4]. On catecholamine stimulation, Plin1 is phosphorylated by cAMP-dependent protein kinase and induces a translocation of hormone-sensitive lipase (HSL) from cytoplasm to lipid droplets [5] and indirectly activates adipose triglyceride lipase (ATGL) [6], hence conferring a full lipolysis response. Plin1 downregulation [7,8] may impair its barrier function and lead to increased lipolysis in the absence of hormonal stimulation [2]. Plin1 deficiency (Plin1-/-) in mice results in low body fat and aberrant lipolysis [9–11], and Plin1 mutations in human cause partial lipodystrophy associated with hyperlipidemia and insulin resistance [12].

Lipodystrophy is a rare disease featured by partial or complete loss of adipose tissue, which is commonly associated with metabolic disturbances such as dyslipidemia, ectopic lipid accumulation and insulin-resistant diabetes, identical to that occurring in obesity [13,14]. Several genetic defects have been identified in different types of human lipodystrophies [13,14]. Mutations in 1-acylglycerol-3-phosphate O-acyltransferase 2 (AGPAT2) [15], Berardinelli-Seip congenital lipodystrophy 2/Seipin [16–18] and caveolin-1 [19] cause congenital complete lipodystrophy. Mutations in lamin A/C [20,21], peroxisome proliferator-activated receptor-γ (PPARγ) [22], fat-specific protein 27-kDa (Fsp27/Cidec) [23] and perilipin 1 (Plin1) [12] lead to partial lipodystrophy. These lipodystrophic genes may express in various cells and execute distinct functions, but each is assumed to involve in the process of lipogenesis or/and adipogenesis [13,14]. To date, the mechanisms of how PPARγ mutation attenuates adipogenesis to confer adipose tissue deficiency is well recognized [24,25]. It has been reported that depletion of AGPAT2 enzyme for triglyceride synthesis inhibits adipogenesis of OP9 bone-marrow stromal cells [26]. Seipin is an endoplasmic membrane protein that may contribute to lipid-droplet organization [27]. Seipin knockdown might perturb adipocyte differentiation of C3H10T1/2 mesenchymal stem cells or 3T3-L1 adipocytes [17,18]. These observations suggest that attenuation in adipocyte differentiation could be a common reason for the lipodystrophy.

To explore the mechanism underlying the lipodystrophic phenotype in mice and humans with Plin1 defect, this study examined the role of Plin1 in adipogenesis and adipocyte differentiation. To avoid the disadvantage of preadipocyte cell lines, we investigated adipocyte development in vivo and adopted primary adipose stromal-vascular cells (SVCs) for adipocyte differentiation in vitro. The results revealed that Plin1 ablation caused aberrant differentiation and development of adipose cells, with lacking the population of adipose progenitor cells capable of adipogenesis in vivo and with dysregulation of adipogenic signaling. We suggest that Plin1 was required for lipid droplet growth and later-stage adipocyte differentiation. Our finding may provide a mechanic explanation for the pathology of partial lipodystrophy in patients with Plin1 defect.

## Methods

### 2.1. Antibodies

Polyclonal antibodies against Plin1, Plin2, or HSL were from C. Londos (the U.S. National Institutes of Health). Anti-Fsp27 antibody was from P. Li (Tsinghua University, Beijing). Other antibodies were from Abcam or Santa Cruz Biotechnology.

### 2.2. Animals

Plin1-/- mice in the 129/SvEv background were from the laboratory of C. Londos (US National Institutes of Health) [9,11], and were housed at Peking University Health Science Center in accordance with the guidelines of the animal care utilization committee of the institute. Plin1-/- male mice and their control wild-type littermates were used for the various experiments. Mice were killed by cervical dislocation after anesthetized with pentobarbital (40 mg/kg, i.p.).

### 2.3. Recombinant adenovirus

Recombinant adenovirus was constructed by use of the AdEasy Adenoviral Vector System (Stratagene) and purified as described [5]. The recombinant adenovirus had 2 separate expression-cassettes that allow for dually expressing green fluorescent protein (GFP) and Plin1 or Plin2 in a single cell. For adenovirus infection, SVCs were cultured overnight and then infected with the adenoviral mixture for 3 h. Then, the infection mixture was removed, and fresh culture medium was added. Cells were cultured for another 2 days and then induced for differentiation.

### 2.4. Morphology analysis

The epididymal and retroperitoneal fat depots were isolated from Plin1-/- mice and wild-type littermates at 8 and 25 weeks of age. Whole-mounted adipose tissue was sectioned at 8-μm thick and stained with hematoxylin and eosin (HE). Fully mature unilocular adipocytes have only a single, large lipid droplet that fills the majority of the cytoplasm. Multilocular adipocytes contain multiple, small lipid droplets in cytoplasm. The number and size of unilocular and multilocular adipocytes were measured and analyzed.

### 2.5. Isolation and differentiation of SVCs into adipocytes

SVCs were isolated from epididymal or inguinal subcutaneous fat pads of Plin1-/- mice and wild-type mice at 8~12 weeks old, according to the method [28] with our modification [8,29,30]. Briefly, the fat pads were minced and digested in serum-free DMEM containing 0.8 mg/ml type I collagenase and 1% defatted BSA, for 40 min at 37°C in a water bath shaken at 120 cycles/min. The digestion mixture was filtered through 80 and 400 steel meshes to remove debris and floating primary adipocytes. The infranatant was the SVCs fraction containing adipose precursor cells, and then collected by centrifugation at 500×g for 20 min for subsequent experiments. For adipocyte differentiation, SVCs were plated and cultured for 2 days in DMEM/F12 (1:1) medium containing 5% fetal bovine serum (FBS). Thereafter, SVCs were differentiated initially (day 0) in a standard adipocyte differentiation cocktail [28] containing 250 μM isobutylmethylxanthine (IBMX), 17 nM insulin, 0.1 μM dexamethasone, and 60 μM indomethacin in DMEM/F12 (1:1) supplemented with 5% FBS. The cocktail was removed after differentiation for 48 h (day 2). Then, cells were induced for an additional 2 days (day 3~4) with 17 nM insulin in 10% FBS-DMEM/F12 and thereafter maintained in 10% FBS-DMEM/F12. For lipid loading, oleic acid was added to the culture at 400 μM as a 5:1 molar complex with FFA-free BSA [31].

### *2.6. Flow* cytometry *analysis of SVC cells*

SVCs were isolated from epididymal adipose tissues in Plin1-/- and wild-type mice as described above and underwent analysis immediately. Cells were stained with a mixture of the following antibodies against adipocyte progenitor markers: CD34-Alexa Fluor 647, CD29-FITC, Sca-1-PE, and CD24-PerCP-Cy5.5. To set proper compensation and population gates, single-color positive cells were stained with each antibody alone, and cells were incubated with isotype-matched IgG labeled with Alexa Fluor 647, FITC, PE or PerCP-Cy5.5 as negative controls. After incubation for 30 min on ice, the cells underwent four-color analysis with Calibur flow cytometry and CellQuest software. All antibodies and instrument used were from BD Biosciences.

### 2.7. Lipid droplet staining with Oil-red O and Nile red

Differentiated adipocytes were fixed for 15 min in 4% paraformaldehyde. Cells were stained with 0.5% Oil-red O in 60% isopropanol for 15 min. After 3 washes with PBS, cells were examined under a light microscope. For fluorescence microscopy, cellular nuclei were stained with Hoechst 33258, then lipid droplets were stained with Nile red for 5 min. The lipid droplets stained with Nile red originally showed yellow/gold fluorescence under a Nikon Eclipse 50i microscope with a 450- to 490-nm excitation filter and 520-nm barrier filter. When the images were merged in red and blue fluorescent channels by use of image processing software supplied by the microscope manufacture, the lipid droplets appeared red and nuclei blue.

### 2.8. Immunofluorescence

Cells were fixed for 20 min with 4% paraformaldehyde and 0.01% Triton X-100 in PBS, followed by 3 rinses with PBS for 5 min each. Nonspecific binding sites in cells were blocked with 5% donkey serum for 60 min. The cells were incubated with polyclonal antibodies against Plin1 or Plin2 [32] at 1:500 overnight at 4°C, then with FITC-conjugated secondary antibody at 1:500 for 1 h. Cell nuclei were stained with Hoechst 33258. Immunofluorescent signals were observed under a Nikon Eclipse 50i microscope.

### 2.9. Immunoblotting and quantitative RT-PCR

Equal amount proteins extracted from adipose tissue or differentiated adipocytes of the mice were underwent SDS-PAGE and immunoblotting analysis with the primary antibodies and horseradish peroxidase-conjugated IgG. The blots were developed with enhanced chemiluminescence (ECL) detection reagents (Applygen Technologies, Beijing). Total RNA was extracted from adipose tissue and differentiating SVCs with use RNAtrip reagent (Applygen Technologies, Beijing) and reverse-transcribed to generate first-strand cDNA [33]. Real-time PCR was carried out in duplicate in an Mx3000 Quantitative PCR System (Stratagene). The relative target mRNA levels were analyzed and normalized to that of internal control 18S rRNA. The primer sets were listed in S1 Table.

### 2.10. Statistics

Data shown are mean ± SEM. The 2-tailed Student's *t* test was used for statistical analysis. P < 0.05 was considered statistically significant.

## Results

### 3.1. Aberrant morphology of adipose cells in Plin1-/- mice

**Fig 1:**
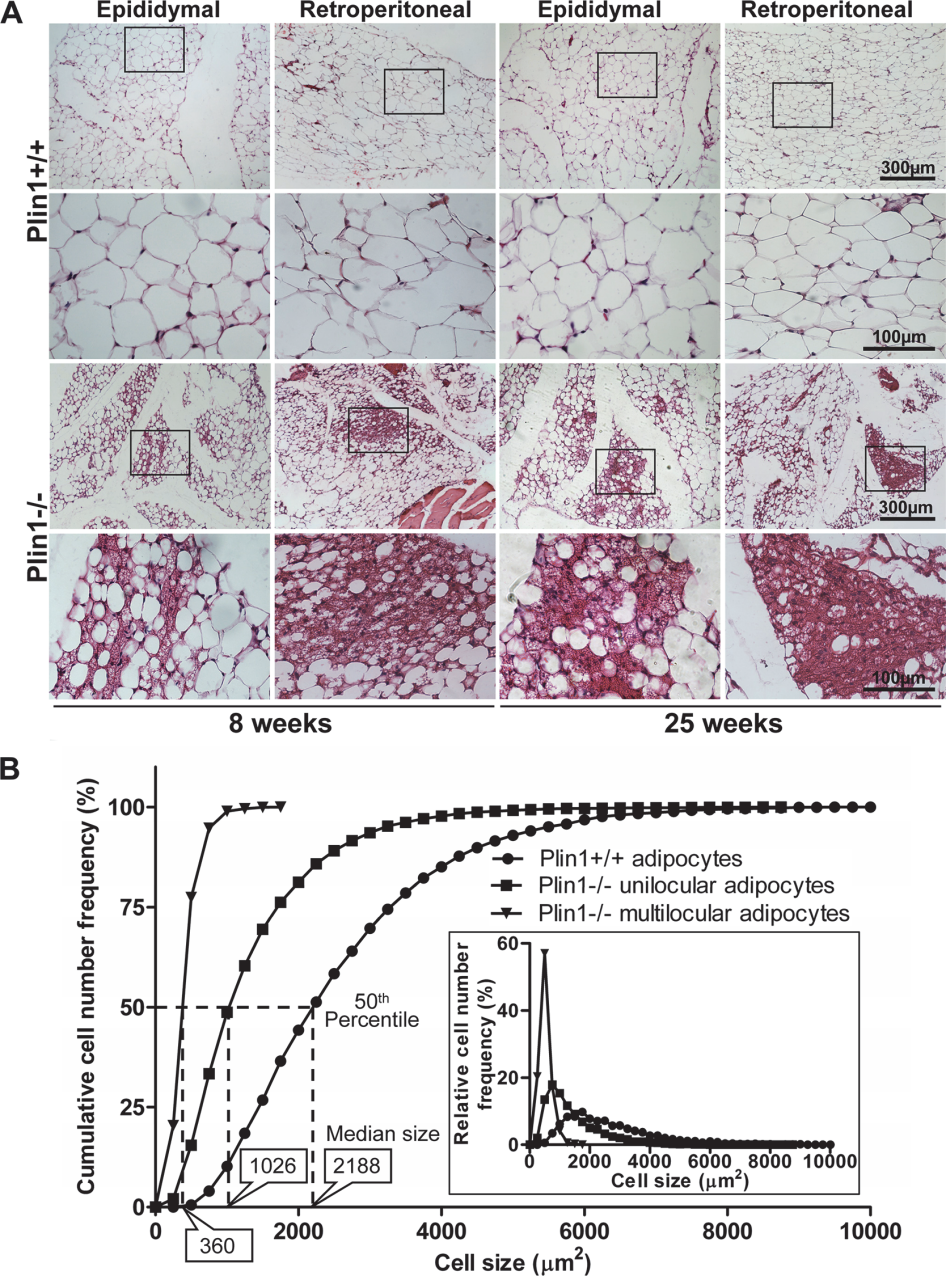
Aberrant morphology of adipose tissue in Plin1-/- mice. (A) Hematoxylin and eosin (HE) staining of adipose tissues in Plin1-/- and Plin1+/+ male mice at 8 and 25 weeks old. The boxed fields (100×) were showed underneath at high magnification (400×). (B) Cumulative adipocyte frequency from epididymal adipose tissues of Plin1-/- and Plin1+/+ mice (n = 3 for each genotype) at age of 25 weeks. For each mice, 10~12 fields of vision at 100 × magnification from different segments of fat tissue were randomly selected for analysis. A total 6,265 of Plin1+/+ unilocular adipocytes (circles) and 11,471 Plin1-/- unilocular (squares) and 1,881 Plin1-/- multilocular (triangles) adipocytes were counted. Cell area was measured by use of NIH Image-J software. The y-axis values represent the cumulative cell percentage for adipocytes at and below the corresponding sizes on the x-axis. The lines labeled as 50th percentile intersect the curves at the median cell sizes (boxes) below which 50% of the adipocytes in each population were distributed. The leftward shift of the curves indicates that the adipocyte population in Plin1-/- mice tend toward smaller cell area as compared with that in Plin1+/+ mice. The inset panel shows the relative (non-cumulative) cell frequency versus cell area, and demonstrates that Plin1-/- mice have a greater proportion of small adipocytes than Plin1+/+ mice.

We examined the morphologic characteristic of the epididymal and retroperitoneal fat tissues in Plin1-/- male mice and wild-type littermates at 8 and 25 weeks old. Adipose tissues in wild-type mice had no multilocular adipocytes but showed closely arranged unilocular adipocytes, each with a single, large lipid droplet that fills the majority of the cytoplasm (Fig. 1A). In contrast, adipose tissues in Plin1-/- mice at 8 weeks old already showed typical histologic characteristics of partial lipodystrophy, as indicated by plenty of small unilocular adipocytes, with multiple small lipid droplets in cytoplasm (Fig. 1A). The total adipocyte population in Plin1-/- adipose tissue consisted of 86% unilocular adipocytes and 14% multilocular adipocytes, whereas Plin1+/+ fat contained only unilocular adipocytes. However, the median cell area of Plin1-/- unilocular adipocytes was 1026 μm^2^, ~50% less than the area (2188 μm^2^) of wild-type adipocytes (Fig. 1B). Plin1-/- multilocular adipocytes were small, with median cell area 360 μm^2^. The trend toward smaller adipocytes in Plin1-/- mice was indicated by leftward shifts of curves for cumulative and relative (inset panel) frequency of cell number distribution versus cell size as compared with the wild type (Fig. 1B).

### 3.2. Dysregulation of adipogenic pathway in adipose tissues in Plin1-/- mice

**Fig 2:**
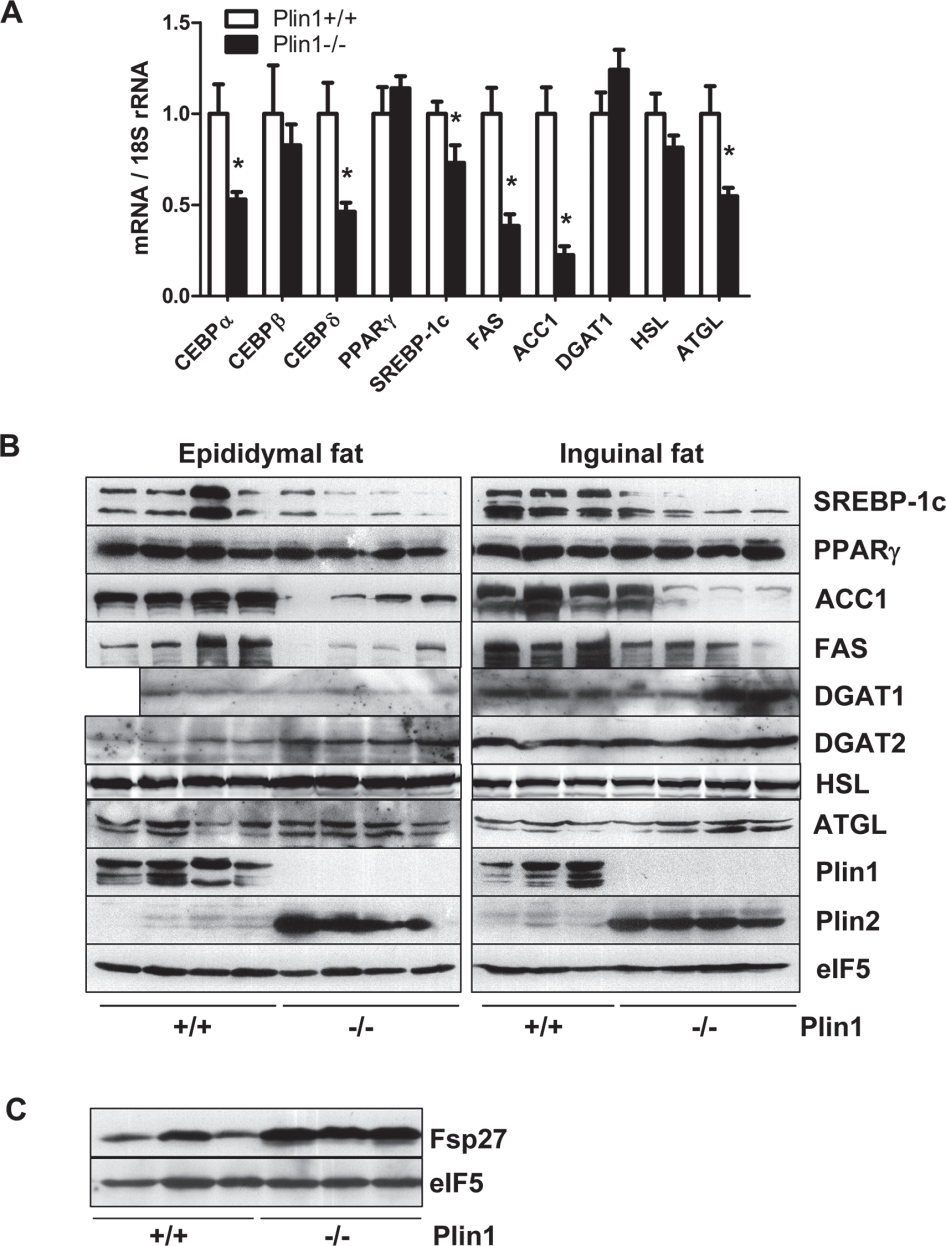
Dysregulation of adipogenic signaling in adipose tissues in Plin1-/- mice. Adipose tissue isolated from Plin1-/- and Plin+/+ mice was utilized for the following examinations. (A) Relative levels of target mRNA detected by real-time PCR. *, P < 0.05 compared with Plin1+/+ mice. (B and C) Immunoblotting.

CCAAT-enhancer-binding proteins (C/EBPs) and PPARγ are the master transcription factors in adipogenesis process [25]. Sterol regulatory element-binding transcription factor 1c (SREBP1c) may also participate in adipocyte gene expression and fat synthesis [25]. In adipose tissue of Plin1-/- mice in contrast to wild-type mice fat, although mRNA and protein expressions of C/EBPβ and PPARγ were unchanged, the mRNA expression of C/EBPα and δ was significantly downregulated (Fig. 2A, B). SREBP1c was downregulated slightly at the transcriptional level but decreased significantly at the translational level. For the downstream enzymes involved in adipogenesis, the mRNA and protein levels of diacylglycerol acyltransferase-1 (DGAT1) and HSL were unchanged, but the mRNA and protein expressions of fatty acid synthase (FAS) and acetyl CoA carboxylase-1 (ACC1) was significantly downregulated (Fig. 2A, B). A discrepancy is the expression of ATGL, whose mRNA level was lower but protein level remained to be normal. Plin2 protein was increased in Plin1-/- adipose tissue (Fig. 2B), due to its substitution for Plin1 at the lipid droplet surface in Plin1-/- adipocytes [5]. Also, protein expressions of Fsp27 was increased in Plin1-/- adipose tissue (Fig. 2C).

### 3.3. Low subpopulation of adipocyte progenitor cells capable of in vivo adipogenesis in Plin1-/- adipose tissue

**Fig 3:**
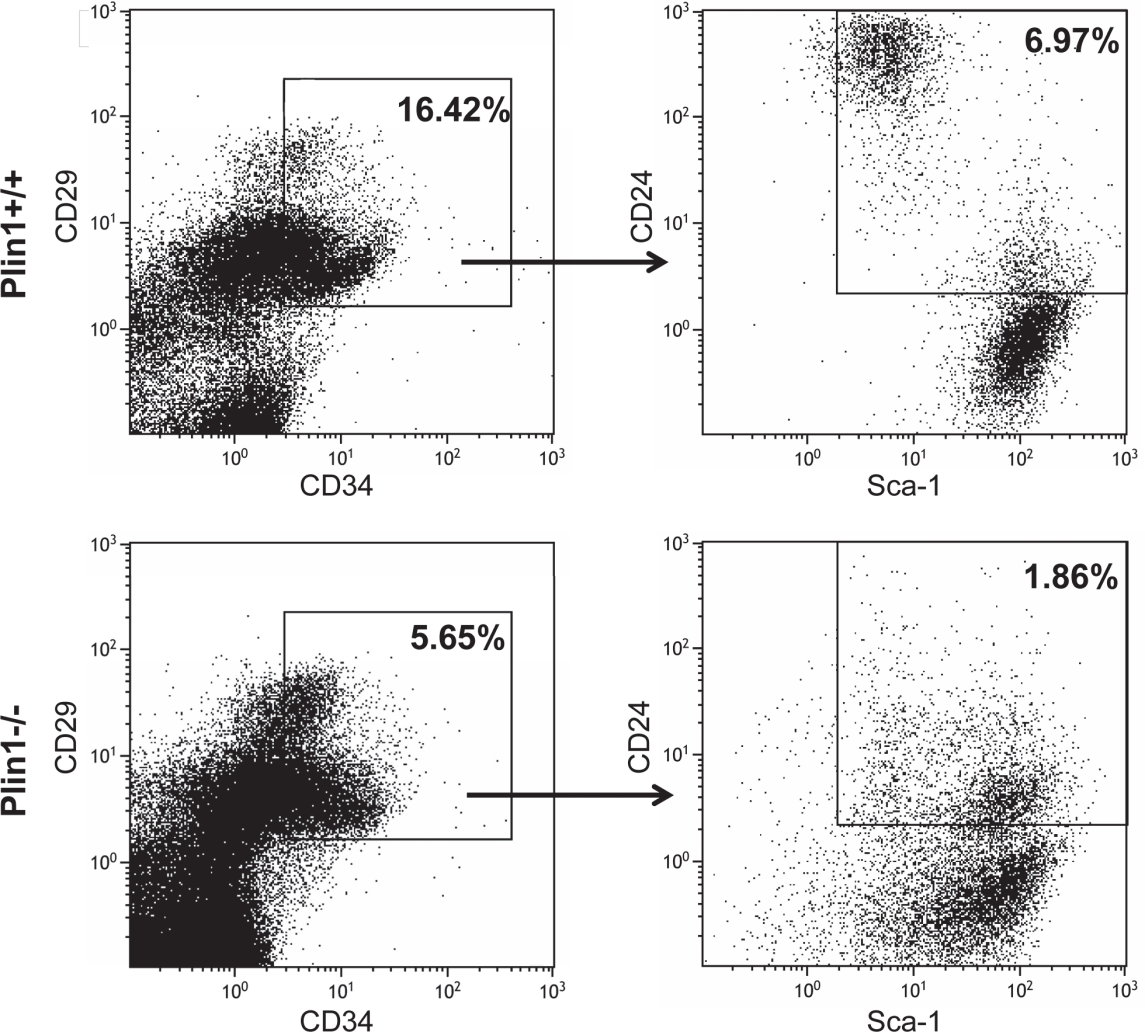
Flow cytometric analysis of SVC cells. The stromal vascular cells (SVCs) were isolated from epididymal fat of Plin1+/+ and Plin1-/- mice and underwent analysis. Adipocyte progenitors in the SVC fraction were immunostained simultaneously with antibodies to CD34 (Alexa Fluor 647), CD29 (FITC), Sca-1 (PE), and CD24 (PerCP-Cy5.5). Cells were incubated with fluorescent isotype-matched IgG as negative controls and single-color positive cells were stained with each antibody alone. The CD29+:CD34+ adipocyte progenitor population was gated (in box) and further separated for the subpopulation coexpressing Sca-1 and CD24 (CD29+:CD34+:Sca-1+:CD24+). The percentage of adipocyte progenitor population in SVC cells was denoted in box.

The SVC fraction consists of a mixed population of cells with variable capacity for adipogenesis. It was recently identified that a subpopulation of adipocyte progenitor cells (Lin-:CD29+:CD34+:Sca-1+:CD24+) in the fraction of SVCs from adipose tissue is capable to differentiate into adipocytes and form adipose depot in vivo in mice [34,35]. We performed real-time PCR to examine the expression of adipose progenitor markers including CD29, CD34, Sca-1 and CD24. The mRNA expression levels of those markers in adipose tissue were lower in Plin1-/- mice than in wild-type mice (S1 Fig.). The flow cytometry analysis revealed that Plin1-/- SVC fraction had low percentage of single-positive cells stained individually with CD29, CD34, or stem cell antigen (Sca-1) (data not shown). In particular, the progenitor cells co-expressing CD29, CD34, Sca-1 and CD24, which represents a subpopulation capable of adipocyte differentiation in vivo, were significantly less (1.86%) in the SVC fraction from Plin1-/- adipose tissue, compared to that (6.97%) in the wild-type SVC preparation (Fig. 3). These data suggest that Plin1-/- SVC cells have low capability of adipocyte differentiation in vivo.

### 3.4. Plin1-/- SVCs fail to differentiate into adipocytes

**Fig 4:**
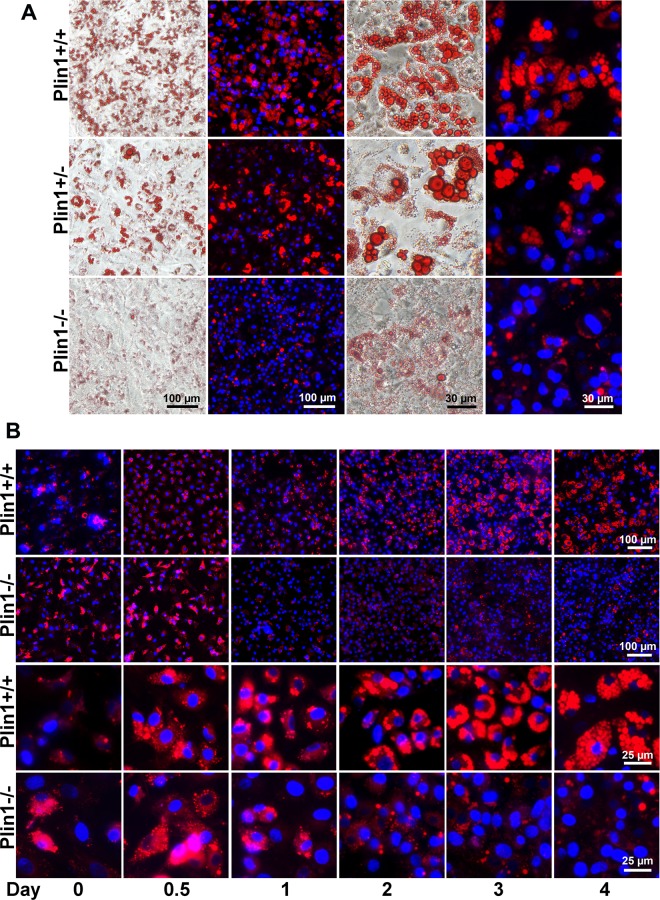
Plin1 defect attenuates the SVC differentiation into adipocytes. (A) Effect of Plin1 gene dosage on adipocyte differentiation. SVCs were isolated from epididymal fat tissue in Plin1+/+, heterozygous Plin1+/-, and Plin1-/- mice, epididymal fat tissue were differentiated into adipocytes. At day 5, intracellular lipid droplets were stained with Oil-red O (red bright-field) and Nile red (red fluorescence). Nuclei (blue) were stained with Hoechst 33258. (B) Dynamic changes of the lipid droplets (red) in Plin1-/- and Plin1+/+ SVCs at day 0, 0.5, 1, 2, 3 and 4 of differentiation.

To examine the affect of Plin1 ablation in adipogenesis, we cultured and differentiated the SVCs to adipocytes. At day 5 after induction, Plin1+/+ SVCs were well differentiated into adipocytes with numerous large lipid droplets stained positively by Oil-red O or Nile red (Fig. 4A). The heterozygous Plin1+/- SVCs were able to generate a few lipid droplets in the size nearly close to that in Plin1+/+ SVCs, but the number of formed droplets was at least ~50% less than that in Plin1+/+ SVCs. By contrast, Plin1-/- SVCs failed to form any large lipid droplet, but had a few moderate-sized droplets and plentiful small or tiny droplets, compared to the heterozygous and wild-type SVCs (Fig. 4A). These results indicate that adipocyte differentiation and adipogenesis are inhibited as consequences of either Plin1 null or Plin1 haploinsufficiency.

Next, we investigated the dynamic changes in lipid droplet development during adipocyte differentiation. SVCs were isolated and cultured for 2 days, then initially differentiated (day 0) with the induction medium containing IBMX, insulin, dexamethasone, and indomethacin. The SVCs were differentiated for 48 h (day 2) and then maintained for another 2 days (day 4) with 17 nM insulin in freshly changed medium. The number and size of lipid droplets in Plin1+/+ SVCs gradually increased, during differentiation. At day 4 after induction, Plin1+/+ SVCs showed numerous large lipid droplets in the cytoplasm (Fig. 4B). Plin1-/- SVCs showed many small or tiny lipid droplets just prior to induction of differentiation (day 0), then these lipid droplets increased in number and became larger during the first 12 h differentiation but thereafter (day 1) appeared diffuse or disappeared. Both the number and size of droplets were significantly decreased in Plin1-/- SVCs as compared with wild-type cells (Fig. 4B). Likely, Plin1-/- SVCs were already enriched with tiny lipid droplets when they were isolated from adipose tissue, but these droplets showed retarded growth during later differentiation.

### 3.5. Dysregulated adipogenic signaling in differentiating Plin1-/- SVCs

**Fig 5:**
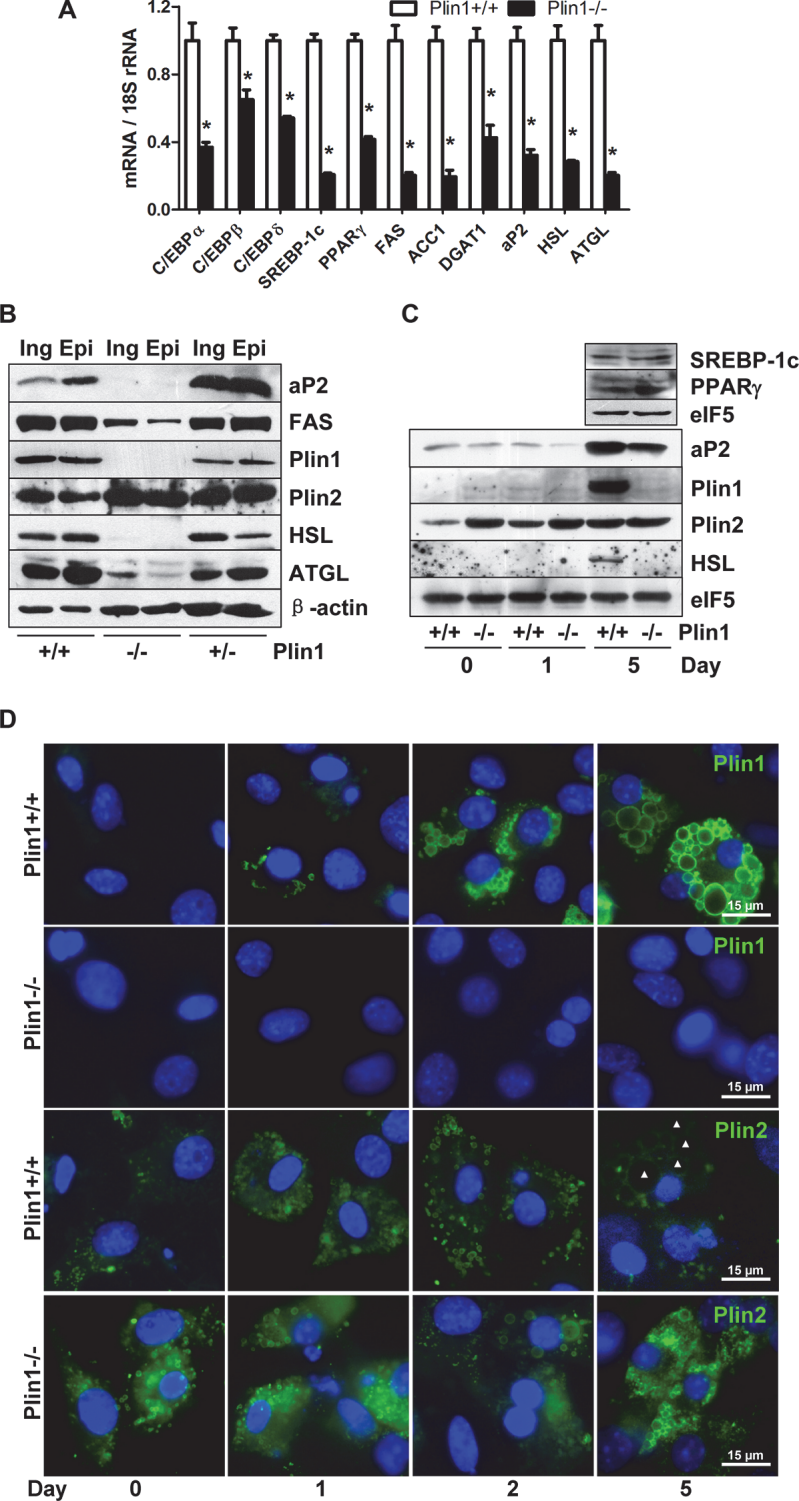
Dysregulation of adipogenic signaling in differentiating Plin1-/- SVCs. The SVC cells isolated from adipose tissues in Plin1+/+, Plin1+/-, and Plin1-/- mice were differentiated. (A) Relative mRNA expression of adipogenic genes at day 5 of differentiation.*, P < 0.05 compared with Plin1+/+ cells. (B) Immunoblotting. Protein expression of adipogenic modulators at day 5 of differentiation in the SVCs from epididymal (Epi) and inguinal (Ing) fat depots in Plin1+/+, Plin1+/-, and Plin1-/- mice. (C) Immunoblotting. Protein expression of adipogenic modulators in the SVCs differentiated for 0, 1, and 5 days. (D) Immunofluorescence of Plin2 and Plin1 switch on lipid droplets. Plin1-/- and Plin1+/+ SVCs were differentiated for 0, 1, 2, and 5 days and immunostained with primary antibody against Plin1 (upper 2 rows) and Plin2 (lower 2 rows) and FITC-conjugated lgG. Nuclei (blue) were stained with Hoechst 33258. Triangles at the central of lipid droplets denote the large droplets disassociated from Plin2 in a fully-differentiated Plin1+/+ adipocyte, compared to the smaller droplets associated with Plin2 in ill-differentiated Plin1-/- SVCs.

In adipogenic state of Plin1-/- SVC differentiation, there were significant decreases in mRNA expression of the transcription factors such as C/EBPs, SREBP1c and PPARγ, and their downstream targets like DGAT1, FAS, ACC1, HSL, ATGL and aP2 (Fig. 5A). Protein levels of PPARγ and SREBP-1c were undetectable at day 1 of differentiation (data not shown) and their expression at day 5 of induction appeared at a similar level in Plin1-/- and wild-type SVCs (Fig. 5C). However, protein expression of HSL, ATGL, and aP2 was downregulated in differentiating Plin1-/- SVCs from either epididymal or inguinal fat pads (Fig. 5B, C). Protein level of FAS was greatly decreased in Plin1-/- SVCs (Fig. 5B). Although heterozygous Plin1+/- SVCs cannot be differentiated fully, these cells with Plin1 haploinsufficiency still expressed near-normal levels of FAS, HSL and ATGL proteins (Fig. 5B).

We had previously observed a heavy accumulation of Plin2 around the lipid droplets in differentiated Plin1-/- mouse embryonic fibroblasts [5]. In differentiated Plin1-/- SVCs, Plin2 protein was significantly increased (Fig. 5B, C). Immunofluorescent staining showed that Plin2 appeared and associated with small lipid droplets both in Plin1-/- and Plin1+/+ SVCs at day 1~2 of induction (Fig. 5D). In Plin1+/+ SVCs, Plin2 signals became dim when the droplets enlarged and recruited Plin1 lately at day 5 of differentiation; however, in Plin1-/- SVCs, Plin2 as a replacement for Plin1 was still greatly assembled on the lipid droplets at day 5, due to the absence of Plin1 on the droplet surface (Fig. 5D).

### 3.6. Exogenous Plin1 expression restores Plin1-/- adipocyte differentiation

**Fig 6:**
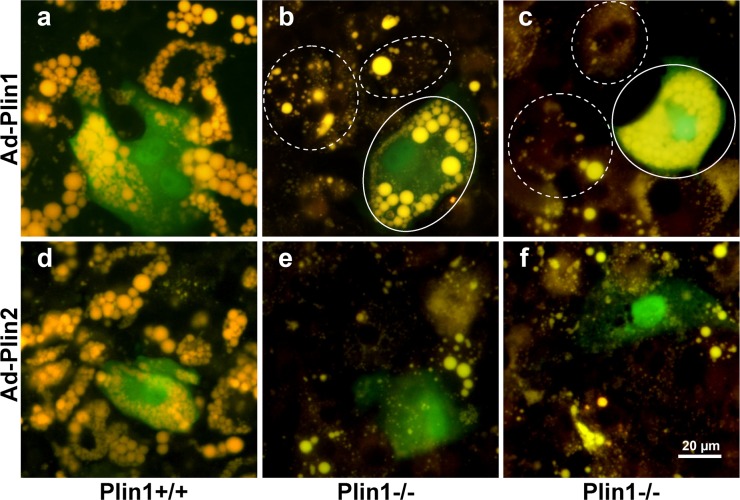
Exogenous Plin1 expression restores Plin1-/- adipocyte differentiation. Plin1-/- and Plin+/+ epididymal SVCs were infected with the adenovirus dually expressing green fluorescence protein (GFP) and Plin1 (Ad-Plin1, a−c), with the adenovirus expressing GFP and Plin2 (Ad-Plin2, d−f) as the control. Fluorescence microscopy of cells induced to differentiate into adipocytes for 4 days, then stained with Nile red. Lipid droplets exhibit yellow/gold fluorescence. GFP-positive cells express ectopic Plin1 or Plin2. Well-differentiated Plin1-/- cells with Ad-Plin1 infection (circles with solid line) and poorly-differentiated cells without Ad-Plin1 infection (circles with dashed line) are labeled in the field of vision (b, c).

Before induction of adipocyte differentiation, isolated SVCs were infected with the recombinant adenovirus that dually expressed GFP and Plin1. Adenovirus dually expressing GFP and Plin2 served as a control. At day 5 of differentiation, intracellular lipid droplets were stained with Nile red. GFP signals indicated that the SVCs were successfully infected. Plin1-/- SVCs infected with Ad-Plin1 showed near-normal adipocyte morphologic features, characterized by many large lipid droplets, but this phenomenon was not observed in the same vision field of Plin1-/- SVCs that were not infected with Ad-Plin1 nor in Plin1-/- SVCs infected with Ad-Plin2 (Fig. 6). Thus, exogenous expression of Plin1 may have restored the defective Plin1-/- adipocyte differentiation.

### 3.7. Enhanced induction improves Plin1-/- adipocyte differentiation

**Fig 7:**
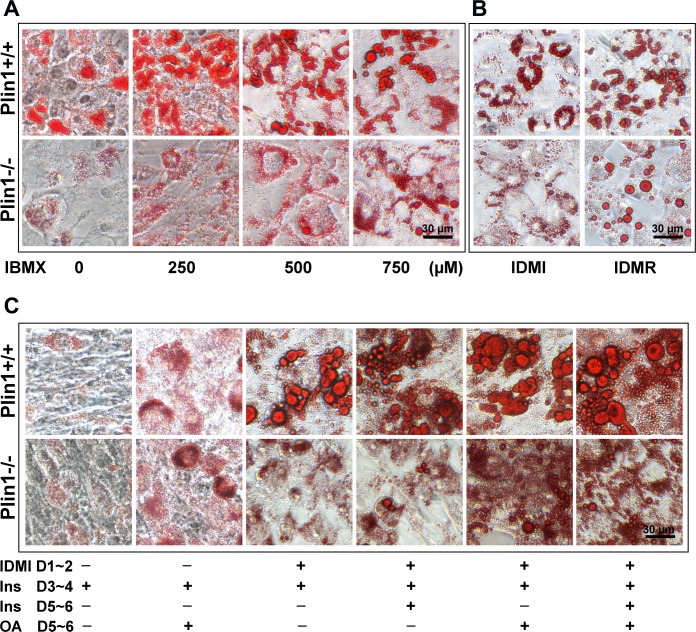
Enhanced induction improves Plin1-/- SVC differentiation. Epididymal SVCs were induced for 2 days (D1~2) in differentiation medium (IDMI) containing 250 μM IBMX, 17 nM insulin, 0.1 μM dexamethasone and 60 μM indomethacin, followed by an additional 2-day (D3~4) treatment with 17 nM insulin. Lipid droplets were stained with Oil-red O. (A) Effect of IBMX dose on lipid-droplet formation and adipocyte differentiation of Plin1+/+ and Plin1-/- SVCs. (B) Effect of replacing indomethacin with 5 μM rosiglitazone (IDMR) on lipid-droplet growth and adipogenesis in SVCs. (C) After day-4 differentiation, SVCs were further stimulated with 17 nM insulin (Ins), 400 μM oleic acid (OA), or both for an additional 2 days (D5~6).

Insulin, IBMX, and PPARγ ligand are common inducers of adipocyte differentiation [28]. We investigated whether enhanced induction could ameliorate defective Plin1-/- adipocyte differentiation. SVCs were induced to differentiate for 2 days with 250 μM IBMX, 17 nM insulin, 0.1 μM dexamethasone, and 60 μM indomethacin, followed by an additional 2-day stimulation with 17 nM insulin [28]. When IBMX concentration was increased to 500 and 750 μM, the lipid droplets in Plin1-/- SVCs gradually increased and became enlarged (Fig. 7A). Although fewer in number, several lipid droplets in Plin1-/- SVCs appeared to be close to the normal droplet size of Plin1+/+ adipocytes when indomethacin in the medium (IDMI) was replaced with PPARγ agonist rosiglitazone (IDMR, Fig. 7B). During day 5~6 of differentiation, the addition of insulin, oleic acid, or both, further increased the number and size of lipid droplets in Plin1-/- SVCs (Fig. 7C). These observations suggest that enhanced induction and fatty acid loading promote lipid droplet growth and ameliorated Plin1-/- adipocyte differentiation.

## Discussion

For in vivo study, Plin1-/- mice at 8 weeks old already showed typical histologic phenotype of lipodystrophy featured by insufficient unilocular adipocytes and aberrant adipocyte morphology. The epididymal adipose tissue in Plin1-/- mice contained ~14% of multilocular adipocytes, with multiple small lipid droplets; the remaining 86% of Plin1-/- adipocytes were unilocular with a single droplet, but their median cell-size was ~50% smaller than that of wild-type adipocytes. Recent studies suggest that a subpopulation of adipocyte progenitors (Lin-:CD29+:CD34+:Sca-1+:CD24+) in the SVC fraction from murine adipose tissue may be capable of differentiating into an adipose depot in vivo [34,35]. The mRNA expression of CD34, CD29, Sca-1 and CD24 in adipose tissue was lower in Plin1-/- mice than in wild-type mice. Consistently, Plin1-/- SVC fraction had less progenitor cells labeled by CD29, CD34, Sca-1 or CD24 alone and contained only a few of CD29+:CD34+:Sca-1+:CD24+ adipose precursor cells, a subpopulation capable of adipogenesis in vivo. To date, the factors influencing adipose progenitor cells are largely unknown. The progenitor proliferation and differentiation might be modulated by the microenvironment signals originating from the microvascular endothelial cells, macrophages and adipocytes in adipose tissue [36,37]. Whether microenvironment factors account for the reduction of adipogenic progenitor subpopulation in Plin1-/- SVCs remains to be further investigated. Low expression of progenitor markers implicates that the early development or growth of the local progenitor pool is likely aberrant in Plin1-/- adipose tissue, leading to a low capability of Plin1-/- SVC differentiation. Also, adipose tissue in Plin1-/- mice showed the downregulation of mRNA and protein expression of adipogenic transcription factors like C/EBPs and SREBP1c, accompanied with the downregulation of lipogenic enzymes like FAS and ACC1. These in vivo data suggest that the impairment of in vivo adipogenesis and lack of adipogenic subpopulation of adipose progenitors might be the basis for impaired adipocyte differentiation and hence lipodystrophic appearance in Plin1-/- mice.

In vitro tests showed that the SVCs from Plin1-/- mice failed to fully differentiate into adipocytes. In general, the transcription factors need to be early activated for controlling the expression of numerous adipogenic genes [24,25]. C/EBPs, SREBP-1c, PPARγ, and the adipogenic genes such as aP2, FAS, HSL and ATGL, were downregulated at the transcriptional level during differentiation of Plin1-/- SVCs. However, the protein expression of PPARγ and SREBP-1c was not altered in Plin1-/- and Plin1+/+ cells, consistent to the similar protein levels of PPARγ between the adipose tissues of Plin1-/- and Plin1+/+ mice. These data implicate that the upstream transcriptional control by PPARγ might remain to be normal during differentiation of Plin1-/- cells. This phenomenon is not a surprise, because Plin1 is a downstream target of PPARγ [38] and thus Plin1 ablation would not reversely affect expression of PPARγ. Likely, the early differentiation process of Plin1-/- SVCs might be initiated by the transcriptional control of PPARγ but then retarded at the terminal stage of adipogenesis. Indeed, several terminal-differentiation factors such as aP2, FAS, HSL and ATGL, were decreased at both mRNA and protein levels during differentiation of Plin1-/- SVCs. FAS catalyzes *de novo* synthesis of fatty acids by conversing acetyl-CoA and malonyl-CoA to palmitate. aP2 is an adipose-specific fatty acid binding protein expressed during adipocyte differentiation and comprises approximately 6% of total adipocyte protein [39]. HSL and ATGL are two major enzymes controlling hydrolysis of triglycerides in adipocytes [5,6]. The significant low expressions of these adipogenic proteins disable synthesis and hydrolysis of adipocyte triglycerides, thus being of reciprocal causation with the failure of adipocyte differentiation.

Prior to induction of differentiation, Plin1-/- SVCs already had numerous tiny lipid droplets, which were able to further enlarge during the first 12-h differentiation but disappear thereafter. The reason for this phenomenon is unclear. Perhaps these Plin1-/- SVCs might derive from a subpopulation of preadipocytes whose differentiation was already initiated in vivo but ceased after the early induction. In adipocytes, growing lipid droplets vary in size and require variable coats of protein compositions, such as Plin1, Plin2 and Fsp27 [32,40–43]. During early differentiation, tiny droplets smaller than 1-μm diameter associate with Plin2 and medium-size (2-μm) droplets associate with both Plin2 and Plin1 [42]. In late differentiation, the droplets larger than 3 μm are coated with only Plin1 [31,42]. We previously revealed that Plin2 is degraded by proteasome, upon the onset of Plin1 [31]. In Plin1-/- SVCs, there was no Plin1 available to replace Plin2 on small lipid droplets, thus lipid droplet growth and later-phase adipocyte differentiation were retarded. Fsp27 is another lipid droplet protein that is relatively selectively expressed in adipocytes. Fsp27 promotes the formation of larger lipid droplets and its deficiency dramatically reduces the size of lipid droplets smaller than 4 μm diameter in adipocytes [43–45]. Interestingly, we observed that the expression of Fsp27 protein was obviously increased in adipose tissue of Plin1-/- mice. We noticed that a number of previous studies actually showed this inverse relationship between expression of Plin1 and Fsp27 [46–50], but this phenomenon seems to be neglected except in the report of Sawada et al [46]. Fsp27 mRNA or protein expression is upregulated upon Plin1 knockout [47] or knockdown [44] but downregulated by Plin1 transgene [46], and vice versa Plin1 mRNA expression is upregulated by FSP27 knockout [48,49] or knockdown [50]. Considering that the both proteins preferentially locate to lipid droplets, we propose that Fsp27 upregulation by Plin1 depletion and vice versa, might be a compensatory action for stabilizing lipid droplets. Recent studies reveal that the interaction between Plin1 and Fsp27 is essential for lipid droplet growth by transferring lipids from small to large droplets [43,45]. However, this cooperation machinery between completely failed in Plin1-/- SVCs, in which no Plin1 was available to mediate the growth of large lipid droplets, in spite of a compensatory increase in Fsp27. The droplet growth in Plin1-/- SVCs were rescued by adenoviral expression of Plin1, confirming the essential role of Plin1 in terminal adipocyte differentiation.

Heterozygous Plin1+/- SVCs can be partially differentiated and fulfilled with a number of lipid droplets, with both the number and size more than in Plin1-/- SVCs but markedly less than in Plin1-/- SVCs. This phenomenon suggests that Plin1 haploinsufficiency inhibit the droplet formation and adipogenesis, providing a cellular basis for the pathology of smaller-than normal adipocytes and partial lipodystrophy in patients with heterozygous Plin1 mutations [12].The differentiation of adipocytes precursors in primary culture is under the influence of many physiological factors [51]. IBMX stimulates adipocyte differentiation by increasing cellular cAMP [51]. For inducing adipocyte differentiation of wild-type murine SVCs, IBMX was optimal at 250 μM but toxic at 500~750 μM. In contrast, IBMX improved Plin1-/- SVC differentiation in a concentration-dependent manner, with an optimal effect at 750 μM. As well, the lipid droplet growth in Plin1-/- SVCs was ameliorated by PPARγ activator and by prolonged exposure to insulin or fatty-acid loading. Therefore, the enhanced adipogenic stimulation can improve lipid-droplet growth and even drive a few Plin1-/- SVCs toward near-normal adipocyte morphology. These observations implicate that some adipose precursor cells in Plin1-/- mice might be capable of regenerative differentiation in response to persistent adipogenic stimulation *in vivo*, which might also contribute to the “partial” rather than complete lipodystrophic phenotype in mammals with Plin1 defects.

In summary, Plin1 was essential for the later-stage differentiation of adipose progenitor cells. Loss of Plin1 impaired the differentiation and development of adipose cells, which might be one of the pathological bases for lipodystrophy in patients with Plin1 mutation.

## Supporting Information

S1 Fig Expression of adipose progenitor markers. (DOC)

S1 Table Primers for quantitative RT-PCR. (DOC)

## References

[1] Kimmel AR, Brasaemle DL, McAndrews-Hill M, Sztalryd C, Londos C (2010) Adoption of PERILIPIN as a unifying nomenclature for the mammalian PAT-family of intracellular lipid storage droplet proteins. J Lipid Res 51: 468–471. 10.1194/jlr.R000034 19638644 PMC2817576

[2] Brasaemle DL (2007) The perilipin family of structural lipid droplet proteins: stabilization of lipid droplets and control of lipolysis. J Lipid Res 48: 2547–2559. 17878492 10.1194/jlr.R700014-JLR200

[3] Greenberg AS, Egan JJ, Wek SA, Garty NB, Blanchette-Mackie EJ, et al (1991) Perilipin, a major hormonally regulated adipocyte-specific phosphoprotein associated with the periphery of lipid storage droplets. J Biol Chem 266: 11341–11346. 2040638

[4] Brasaemle DL, Rubin B, Harten IA, Gruia-Gray J, Kimmel AR, et al (2000) Perilipin A increases triacylglycerol storage by decreasing the rate of triacylglycerol hydrolysis. J Biol Chem 275: 38486–38493. 10948207 10.1074/jbc.M007322200

[5] Sztalryd C, Xu G, Dorward H, Tansey JT, Contreras JA, et al (2003) Perilipin A is essential for the translocation of hormone-sensitive lipase during lipolytic activation. J Cell Biol 161: 1093–1103. 12810697 10.1083/jcb.200210169 PMC2172984

[6] Granneman JG, Moore HP, Krishnamoorthy R, Rathod M (2009) Perilipin controls lipolysis by regulating the interactions of AB-hydrolase containing 5 (Abhd5) and adipose triglyceride lipase (Atgl). J Biol Chem 284: 34538–34544. 10.1074/jbc.M109.068478 19850935 PMC2787315

[7] Zu L, Jiang H, He J, Xu C, Pu S, et al (2008) Salicylate blocks lipolytic actions of tumor necrosis factor-α in primary rat adipocytes. Mol Pharmacol 73: 215–223. 17911533 10.1124/mol.107.039479

[8] Zu L, He J, Jiang H, Xu C, Pu S, et al (2009) Bacterial endotoxin stimulates adipose lipolysis via toll-like receptor 4 and extracellular signal-regulated kinase pathway. J Biol Chem 284: 5915–5926. 10.1074/jbc.M807852200 19122198

[9] Tansey JT, Sztalryd C, Gruia-Gray J, Roush DL, Zee JV, et al (2001) Perilipin ablation results in a lean mouse with aberrant adipocyte lipolysis, enhanced leptin production, and resistance to diet-induced obesity. Proc Natl Acad Sci USA 98: 6494–6499. 11371650 10.1073/pnas.101042998 PMC33496

[10] Martinez-Botas J, Anderson JB, Tessier D, Lapillonne A, Chang BH, et al (2000) Absence of perilipin results in leanness and reverses obesity in Lepr(db/db) mice. Nat Genet 26: 474–479. 11101849 10.1038/82630

[11] Zhai W, Xu C, Ling Y, Liu S, Deng J, et al (2010) Increased lipolysis in adipose tissues is associated with elevation of systemic free fatty acids and insulin resistance in perilipin null mice. Horm Metab Res 42: 247–253. 10.1055/s-0029-1243599 20091459

[12] Gandotra S, Le Dour C, Bottomley W, Cervera P, Giral P, et al (2011) Perilipin deficiency and autosomal dominant partial lipodystrophy. N Engl J Med 364: 740–748. 10.1056/NEJMoa1007487 21345103 PMC3773916

[13] Garg A (2006) Adipose tissue dysfunction in obesity and lipodystrophy. Clin Cornerstone 8 Suppl 4: S7–S13. 17208666 10.1016/s1098-3597(06)80039-6

[14] Garg A, Agarwal AK (2009) Lipodystrophies: disorders of adipose tissue biology. Biochim Biophys Acta 1791: 507–513. 10.1016/j.bbalip.2008.12.014 19162222 PMC2693450

[15] Agarwal AK, Arioglu E, De Almeida S, Akkoc N, Taylor SI, et al (2002) AGPAT2 is mutated in congenital generalized lipodystrophy linked to chromosome 9q34. Nat Genet 31: 21–23. 11967537 10.1038/ng880

[16] Cui X, Wang Y, Tang Y, Liu Y, Zhao L, et al (2011) Seipin ablation in mice results in severe generalized lipodystrophy. Hum Mol Genet 20: 3022–3030. 10.1093/hmg/ddr205 21551454

[17] Payne VA, Grimsey N, Tuthill A, Virtue S, Gray SL, et al (2008) The human lipodystrophy gene BSCL2/seipin may be essential for normal adipocyte differentiation. Diabetes 57: 2055–2060. 10.2337/db08-0184 18458148 PMC2494687

[18] Chen W, Yechoor VK, Chang BH, Li MV, March KL, et al (2009) The human lipodystrophy gene product Berardinelli-Seip congenital lipodystrophy 2/seipin plays a key role in adipocyte differentiation. Endocrinology 150: 4552–4561. 10.1210/en.2009-0236 19574402 PMC2754678

[19] Kim CA, Delepine M, Boutet E, El Mourabit H, Le Lay S, et al (2008) Association of a homozygous nonsense caveolin-1 mutation with Berardinelli-Seip congenital lipodystrophy. J Clin Endocrinol Metab 93: 1129–1134. 10.1210/jc.2007-1328 18211975

[20] Cao H, Hegele RA (2000) Nuclear lamin A/C R482Q mutation in canadian kindreds with Dunnigan-type familial partial lipodystrophy. Hum Mol Genet 9: 109–112. 10587585 10.1093/hmg/9.1.109

[21] Boguslavsky RL, Stewart CL, Worman HJ (2006) Nuclear lamin A inhibits adipocyte differentiation: implications for Dunnigan-type familial partial lipodystrophy. Hum Mol Genet 15: 653–663. 16415042 10.1093/hmg/ddi480

[22] Hegele RA, Cao H, Frankowski C, Mathews ST, Leff T (2002) PPARG F388L, a transactivation-deficient mutant, in familial partial lipodystrophy. Diabetes 51: 3586–3590. 12453919 10.2337/diabetes.51.12.3586

[23] Rubio-Cabezas O, Puri V, Murano I, Saudek V, Semple RK, et al (2009) Partial lipodystrophy and insulin resistant diabetes in a patient with a homozygous nonsense mutation in CIDEC. EMBO Mol Med 1: 280–287. 10.1002/emmm.200900037 20049731 PMC2891108

[24] Rosen ED, Sarraf P, Troy AE, Bradwin G, Moore K, et al (1999) PPAR gamma is required for the differentiation of adipose tissue in vivo and in vitro. Mol Cell 4: 611–617. 10549292 10.1016/s1097-2765(00)80211-7

[25] Lefterova MI, Lazar MA (2009) New developments in adipogenesis. Trends Endocrinol Metab 20: 107–114. 10.1016/j.tem.2008.11.005 19269847

[26] Gale SE, Frolov A, Han X, Bickel PE, Cao L, et al (2006) A regulatory role for 1-acylglycerol-3-phosphate-O-acyltransferase 2 in adipocyte differentiation. J Biol Chem 281: 11082–11089. 16495223 10.1074/jbc.M509612200

[27] Fei W, Du X, Yang H (2011) Seipin, adipogenesis and lipid droplets. Trends Endocrinol Metab 22: 204–210. 10.1016/j.tem.2011.02.004 21497513

[28] Hausman DB, Park HJ, Hausman GJ (2008) Isolation and culture of preadipocytes from rodent white adipose tissue. Methods Mol Biol 456: 201–219. 10.1007/978-1-59745-245-8_15 18516563

[29] Jiang H, He J, Pu S, Tang C, Xu G (2007) Heat shock protein 70 is translocated to lipid droplets in rat adipocytes upon heat stimulation. Biochim Biophys Acta 1771: 66–74. 17175194 10.1016/j.bbalip.2006.10.004

[30] He J, Jiang H, Tansey JT, Tang C, Pu S, et al (2006) Calyculin and okadaic acid promote perilipin phosphorylation and increase lipolysis in primary rat adipocytes. Biochim Biophys Acta 1761: 247–255. 16545598 10.1016/j.bbalip.2006.02.001

[31] Xu G, Sztalryd C, Lu X, Tansey JT, Gan JW, et al (2005) Post-translational regulation of adipose differentiation-related protein by the ubiquitin/proteasome pathway. J Biol Chem 280: 42841–42847. 16115879 10.1074/jbc.M506569200

[32] Brasaemle DL, Barber T, Wolins NE, Serrero G, Blanchette-Mackie EJ, et al (1997) Adipose differentiation-related protein is an ubiquitously expressed lipid storage droplet-associated protein. J Lipid Res 38: 2249–2263. 9392423

[33] Xu C, He J, Jiang H, Zu L, Zhai W, et al (2009) Direct effect of glucocorticoids on lipolysis in adipocytes. Mol Endocrinol 23: 1161–1170. 10.1210/me.2008-0464 19443609 PMC5419195

[34] Rodeheffer MS, Birsoy K, Friedman JM (2008) Identification of white adipocyte progenitor cells in vivo. Cell 135: 240–249. 10.1016/j.cell.2008.09.036 18835024

[35] Berry R, Rodeheffer MS (2013) Characterization of the adipocyte cellular lineage in vivo. Nat Cell Biol 15: 302–308. 10.1038/ncb2696 23434825 PMC3721064

[36] Maumus M, Sengenes C, Decaunes P, Zakaroff-Girard A, Bourlier V, et al (2008) Evidence of in situ proliferation of adult adipose tissue-derived progenitor cells: influence of fat mass microenvironment and growth. J Clin Endocrinol Metab 93: 4098–4106. 10.1210/jc.2008-0044 18682517

[37] Hutley LJ, Herington AC, Shurety W, Cheung C, Vesey DA, et al (2001) Human adipose tissue endothelial cells promote preadipocyte proliferation. Am J Physiol Endocrinol Metab 281: E1037–1044. 11595661 10.1152/ajpendo.2001.281.5.E1037

[38] Arimura N, Horiba T, Imagawa M, Shimizu M, Sato R (2004) The peroxisome proliferator-activated receptor gamma regulates expression of the perilipin gene in adipocytes. J Biol Chem 279: 10070–10076. 14704148 10.1074/jbc.M308522200

[39] Spiegelman BM, Green H (1980) Control of specific protein biosynthesis during the adipose conversion of 3T3 cells. J Biol Chem 255: 8811–8818. 6773950

[40] Wang SM, Hwang RD, Greenberg AS, Yeo HL (2003) Temporal and spatial assembly of lipid droplet-associated proteins in 3T3-L1 preadipocytes. Histochem Cell Biol 120: 285–292. 14574583 10.1007/s00418-003-0575-7

[41] Wolins NE, Quaynor BK, Skinner JR, Schoenfish MJ, Tzekov A, et al (2005) S3–12, Adipophilin, and TIP47 package lipid in adipocytes. J Biol Chem 280: 19146–19155. 15731108 10.1074/jbc.M500978200

[42] Nagayama M, Uchida T, Gohara K (2007) Temporal and spatial variations of lipid droplets during adipocyte division and differentiation. J Lipid Res 48: 9–18. 17057226 10.1194/jlr.M600155-JLR200

[43] Sun Z, Gong J, Wu H, Xu W, Wu L, et al (2013) Perilipin1 promotes unilocular lipid droplet formation through the activation of Fsp27 in adipocytes. Nat Commun 4: 1594 10.1038/ncomms2581 23481402 PMC3615468

[44] Nishino N, Tamori Y, Tateya S, Kawaguchi T, Shibakusa T, et al (2008) FSP27 contributes to efficient energy storage in murine white adipocytes by promoting the formation of unilocular lipid droplets. J Clin Invest 118: 2808–2821. 10.1172/JCI34090 18654663 PMC2483680

[45] Gong J, Sun Z, Wu L, Xu W, Schieber N, et al (2011) Fsp27 promotes lipid droplet growth by lipid exchange and transfer at lipid droplet contact sites. J Cell Biol 195: 953–963. 10.1083/jcb.201104142 22144693 PMC3241734

[46] Sawada T, Miyoshi H, Shimada K, Suzuki A, Okamatsu-Ogura Y, et al (2010) Perilipin overexpression in white adipose tissue induces a brown fat-like phenotype. PLoS One 5: e14006 10.1371/journal.pone.0014006 21103377 PMC2982838

[47] Castro-Chavez F, Yechoor VK, Saha PK, Martinez-Botas J, Wooten EC, et al (2003) Coordinated upregulation of oxidative pathways and downregulation of lipid biosynthesis underlie obesity resistance in perilipin knockout mice: a microarray gene expression profile. Diabetes 52: 2666–2674. 14578284 10.2337/diabetes.52.11.2666

[48] Toh SY, Gong J, Du G, Li JZ, Yang S, et al (2008) Up-regulation of mitochondrial activity and acquirement of brown adipose tissue-like property in the white adipose tissue of fsp27 deficient mice. PLoS One 3: e2890 10.1371/journal.pone.0002890 18682832 PMC2483355

[49] Li D, Zhang Y, Xu L, Zhou L, Wang Y, et al (2010) Regulation of gene expression by FSP27 in white and brown adipose tissue. BMC Genomics 11: 446 10.1186/1471-2164-11-446 20649970 PMC3091643

[50] Puri V, Virbasius JV, Guilherme A, Czech MP (2008) RNAi screens reveal novel metabolic regulators: RIP140, MAP4k4 and the lipid droplet associated fat specific protein (FSP) 27. Acta Physiol (Oxf) 192: 103–115. 10.1111/j.1748-1716.2007.01786.x 18171433 PMC2880506

[51] Wiederer O, Loffler G (1987) Hormonal regulation of the differentiation of rat adipocyte precursor cells in primary culture. J Lipid Res 28: 649–658. 2440970

