# Supplementary material for: Defective Differentiation of Adipose Precursor Cells from Lipodystrophic Mice Lacking Perilipin 1
Source: PLoS One. 2015 Feb 19;10(2):e0117536. doi: 10.1371/journal.pone.0117536 (PMC4335001; doi:10.1371/journal.pone.0117536)

Supporting Information

**Figure S1. Expression of adipose progenitor markers.** The mRNA expression of adipose progenitor markers including CD29, CD34, Sca-1 and CD24 in adipose tissues from Plin1-/- and Plin+/+ mice.


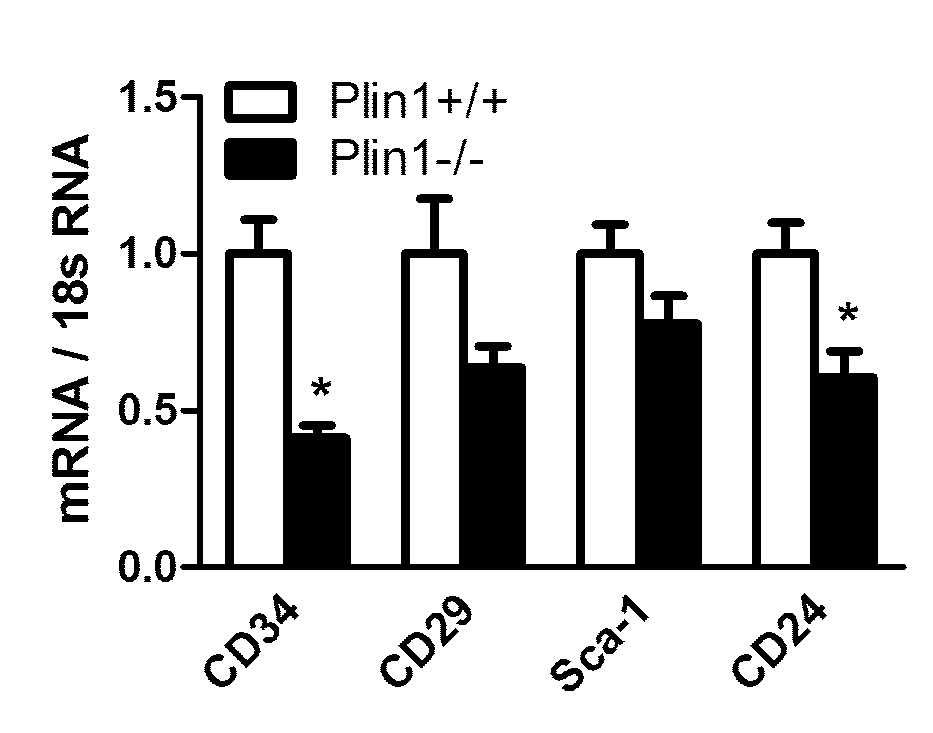

Supplement: S1 Fig — (DOC) [file pone.0117536.s001.doc]
