# Supplementary material for: Defective Differentiation of Adipose Precursor Cells from Lipodystrophic Mice Lacking Perilipin 1
Source: PLoS One. 2015 Feb 19;10(2):e0117536. doi: 10.1371/journal.pone.0117536 (PMC4335001; doi:10.1371/journal.pone.0117536)
Supplement: S1 Table — (DOC) [file pone.0117536.s002.doc]

Supporting Information

**Table S1. Primers for quantitative RT-PCR.** The forward (F) and reverse (R) primers used for quantitative RT-PCR were listed in Table S1.

| **Genes** | **Direction** | **Primer Sequence (5’-3’)** |
| --- | --- | --- |
| aP2 | Forward | GCGTGGAATTCGATGAAATCA |
|  | Reverse | CCCGCCATCTAGGGTTATGA |
| ACC1 | Forward | CAA ATTCTGCTGGAGAAGCC |
|  | Reverse | TACAGGATGGTTTGGCCTTT |
| ATGL | Forward | AACACCAGCATCCAGTTCAA |
|  | Reverse | GGTTCAGTAGGCCATTCCTC |
| CD24 | Forward | CGTTACTTGGATTTGGGGAA |
|  | Reverse | GCTTCTGGCACTGCTCCTAC |
| CD29 | Forward | CAGGAAACCAGTTGCAAATTC |
|  | Reverse | ACACCGACCCGAGACCCT |
| CD34 | Forward | TGGTAGGAACTGATGGGGAT |
|  | Reverse | TGGGTAGCTCTCTGCCTGAT |
| CEBPα | Forward | TGGACA AGA ACAGCA ACGAG |
|  | Reverse | TCACTGGTCAACTCCAGCA |
| CEBPβ | Forward | GAGCGACGAGTACAAGATGCGG |
|  | Reverse | TTGTGCTGCGTCTCCAGGTTG |
| CEBPδ | Forward | CTGAACGACCTATACCTCAGACC |
|  | Reverse | AGCTTCTCTCGCAGTCCAGT |
| DGAT1 | Forward | TTCCGCCTCTGGGCATT |
|  | Reverse | AGAATCGGCCCACAATCCA |
| FAS | Forward | TGGGTTCTAGCCAGCAGAGT |
|  | Reverse | ACCACCAGAGACCGTTATGC |
| HSL | Forward | ACCGAGACAGGCCTCAGTGTG |
|  | Reverse | GAATCGGCCACCGGTAAAGAG |
| PPARγ | Forward | GTTTGCTGTGAAGTTCAATGC |
|  | Reverse | GTCTGTCTCCGTCTTCTTGAT |
| Sca-1 | Forward | ACAGCACAGGCAGGA AGACT |
|  | Reverse | TCGGTATTATCTTCGGGGC |
| SREBP-1c | Forward | TGCTGTTGGCATCCTGCTATC |
|  | Reverse | AGGAGCATCTGCTGGCAGTC |
| 18S rRNA | Forward | GAAACGGCTACCACATCCAAGG |
|  | Reverse | GCCCTCCAATGGATCCTCGTTA |
